# Smartphone Ownership and Interest in Mobile Health Technologies for Self-care Among Patients With Chronic Heart Failure: Cross-sectional Survey Study

**DOI:** 10.2196/31982

**Published:** 2022-01-14

**Authors:** Jonathan W Leigh, Ben S Gerber, Christopher P Gans, Mayank M Kansal, Spyros Kitsiou

**Affiliations:** 1 Department of Biomedical and Health Information Sciences College of Applied Health Sciences University of Illinois at Chicago Chicago, IL United States; 2 Division of Cardiology Department of Medicine University of Illinois at Chicago Chicago, IL United States; 3 Department of Population and Quantitative Health Sciences University of Massachusetts Medical School Worchester, MA United States

**Keywords:** mHealth, smartphone, mobile phone, heart failure, self-care, self-management

## Abstract

**Background:**

Heart failure (HF) is a highly prevalent chronic condition that places a substantial burden on patients, families, and health care systems worldwide. Recent advances in mobile health (mHealth) technologies offer great opportunities for supporting many aspects of HF self-care. There is a need to better understand patients’ adoption of and interest in using mHealth for self-monitoring and management of HF symptoms.

**Objective:**

The purpose of this study is to assess smartphone ownership and patient attitudes toward using mHealth technologies for HF self-care in a predominantly minority population in an urban clinical setting.

**Methods:**

We conducted a cross-sectional survey of adult outpatients (aged ≥18 years) at an academic outpatient HF clinic in the Midwest. The survey comprised 34 questions assessing patient demographics, ownership of smartphones and other mHealth devices, frequently used smartphone features, use of mHealth apps, and interest in using mHealth technologies for vital sign and HF symptom self-monitoring and management.

**Results:**

A total of 144 patients were approached, of which 100 (69.4%) participated in the study (63/100, 63% women). The participants had a mean age of 61.3 (SD 12.25) years and were predominantly Black or African American (61/100, 61%) and Hispanic or Latino (18/100, 18%). Almost all participants (93/100, 93%) owned a cell phone. The share of patients who owned a smartphone was 68% (68/100). Racial and ethnic minorities that identified as Black or African American or Hispanic or Latino reported higher smartphone ownership rates compared with White patients with HF (45/61, 74% Black or African American and 11/18, 61% Hispanic or Latino vs 9/17, 53% White). There was a moderate and statistically significant association between smartphone ownership and age (Cramér *V* [Φ_C_]=0.35; *P*<.001), education (Φ_C_=0.29; *P*=.001), and employment status (Φ_C_=0.3; *P*=.01). The most common smartphone features used by the participants were SMS text messaging (51/68, 75%), internet browsing (43/68, 63%), and mobile apps (41/68, 60%). The use of mHealth apps and wearable activity trackers (eg, Fitbits) for self-monitoring of HF-related parameters was low (15/68, 22% and 15/100, 15%, respectively). The most popular HF-related self-care measures participants would like to monitor using mHealth technologies were physical activity (46/68, 68%), blood pressure (44/68, 65%), and medication use (40/68, 59%).

**Conclusions:**

Most patients with HF have smartphones and are interested in using commercial mHealth apps and connected health devices to self-monitor their condition. Thus, there is a great opportunity to capitalize on the high smartphone ownership among racial and ethnic minority patients to increase reach and enhance HF self-management through mHealth interventions.

## Introduction

Heart failure (HF) is a serious cardiovascular condition associated with substantial morbidity, mortality, and health care costs. In the United States, the estimated prevalence of HF is 6.5 million, and it is expected to increase to nearly 8.5 million by 2030 owing to the rapid growth of the aging population [1]. Despite improvements in patient outcomes with pharmacological therapy and surgical treatment, the clinical burden of HF remains high [2]. Approximately 25% of patients are readmitted to the hospital within 30 days, and up to 50% are readmitted within 6 months following HF-related hospitalization [3-5]. This results in significant, potentially avoidable costs for our already strained health care system [6,7]. In 2020, the direct and indirect costs of HF management were estimated to be US $43.6 billion [8]. Of these costs, 70% were attributed to direct medical expenditures because of hospitalizations for acute decompensated HF [8].

A large portion of the health care use costs and many HF-related deaths can potentially be prevented if patients consistently engage in effective self-care [9,10]. Evidence from clinical trials and systematic reviews shows that patients who adhere to their treatment plan and are actively engaged in their own care are more likely to have improved survival, decreased hospital readmission rates, and better quality of life [11-14]. HF self-care is defined as a naturalistic decision-making process comprised of 3 pillars: self-care maintenance, self-care monitoring, and self-care management. Self-care maintenance involves the choice of behaviors that maintain physiological stability, such as taking medications as prescribed, following a sodium-restricted diet, engaging regularly in physical activity or exercise, receiving an influenza vaccine or other necessary vaccinations, quitting smoking and drinking, and attending routine health care appointments. Self-care monitoring involves regular weighing and monitoring of vital signs and symptoms (eg, blood pressure, shortness of breath, and edema) as well as recognition and interpretation of these symptoms. Self-care management refers to the actions taken by the patient in response to HF symptoms and possible deterioration (eg, taking an extra diuretic dose and contacting the HF care team) and to the evaluation of the response to the treatment implemented. Clinical guidelines stress the importance of effective HF self-care as part of a successful treatment [10,15,16]. However, HF self-care is poor among patients with HF [9], especially minorities and people of low socioeconomic status [17,18]. Previous studies have shown that lack of adherence to prescribed medications, sodium-restricted diet, and self-monitoring of symptoms and weight is particularly high, with most studies citing rates of approximately 40% to 60% [19-22].

Rapidly evolving mobile health (mHealth) technologies such as smartphones, mHealth apps, SMS text messaging, wearable activity tracking devices, and other smart and connected health technologies have become increasingly ubiquitous, offering great opportunities for supporting many aspects of HF self-care. Through real-time collection and visualization of patient-generated health data, mHealth apps and connected health devices can enhance self-monitoring of HF signs and symptoms (eg, heart rate, weight, pulse oximetry, and blood pressure) and help patients become more aware of how their bodies work and what is normal and be alerted to health changes that may require medical attention. Furthermore, mHealth apps have the potential to improve self-care adherence through a variety of functionalities and behavior change tools such as medication reminders, behavioral prompts, electronic diaries, personalized feedback, patient education, and social networking [23]. SMS text messaging is another powerful mHealth tool that can be used to deliver behavior change interventions to improve self-management of HF [24,25] and other chronic diseases [26]. SMS text messaging is low-cost, does not require great technological expertise, is increasingly used by people from all socioeconomic classes and age groups [26], and has the advantage of being asynchronous (ie, it can be delivered as soon as the phone is turned back on and accessed by the recipient at any time). Several systematic reviews and meta-analyses have demonstrated the efficacy of mHealth interventions for self-management of long-term conditions (eg, HF [27,28], diabetes [29,30], and hypertension [31]). In the area of HF, a recent meta-analysis found that mHealth interventions involving data transmission to a clinical care team and a feedback loop to the patient can lead to significant reductions in all-cause mortality and HF-related hospitalizations and improve HF self-care and health-related quality of life [27]. However, it is important to note that not all mHealth technologies and interventions are effective. A Cochrane review of mHealth-delivered educational interventions for people with HF found evidence of little to no improvement in HF-related hospitalizations and self-care compared with usual care [32].

Given the increasing interest in the potential of mHealth technologies to improve self-care and reduce hospitalizations in patients with HF, there is a critical need to better understand the adoption and attitudes of patients with HF toward the use of commercial mHealth technologies for self-monitoring and management of HF symptoms—especially among racial and ethnic minority patients (eg, Black or African American and Hispanic or Latino), who have the highest incidence of HF across all age groups [1], and female patients, who have been underrepresented in HF research [33]. Previous mHealth trials [27] and survey studies in HF [34-36] have included primarily White, male patients and focused on *smart devices* in general [34] or smartphone ownership only [35], overlooking adoption of other important mobile technologies such as SMS text messaging and wearable activity trackers that can play an important role in self-monitoring and delivery of behavior change interventions to support HF self-care. We used a holistic approach to develop an mHealth questionnaire based on a review of previous studies and input from patients and clinicians to address this gap. Subsequently, we conducted a survey in a predominantly racial and ethnic minority HF population in an urban academic health care setting to assess patients’ attitudes toward the use of smartphones, SMS text messaging, wearable devices, and other mHealth technologies that support functions of HF self-care. We also explore the relationship between mHealth adoption and socioeconomic characteristics, including educational status.

## Methods

### Study Design

The study was a single-site, cross-sectional survey of adult outpatients conducted between June and September 2018 at the outpatient HF clinic at the University of Illinois Hospital and Health Sciences System. Human research ethics approval was received from the University of Illinois, Chicago Institutional Review Board (research protocol 2017-0395).

### Eligibility Criteria

Adults (aged ≥18 years) with a diagnosis of HF (confirmed by the attending cardiologist based on clinical evaluation and transthoracic echocardiography), able to speak English, and willing to participate in the study were eligible to participate in the survey. The exclusion criteria were patients with a diagnosis of major cognitive impairment (ie, dementia) documented in their medical records.

### Survey Development

The cross-sectional survey used in this study (Multimedia Appendix 1) was designed based on a review of other mHealth surveys [37-40] and comprised 34 items across 4 sections. The first section (8 items) included questions about mobile phone ownership, use of SMS text messaging, and interest in receiving SMS text messages for HF self-care. The second section (10 items) included questions in relation to smartphone ownership and use, internet access and data plans, frequently used smartphone features, use of mHealth apps, and interest in specific smartphone app features for HF self-care. The third section (9 items) included questions in relation to ownership of other mobile and connected health technologies (eg, tablets and wearable activity tracking devices) as well as interest in using mHealth devices for HF self-monitoring and transmitting this information to their physician. The fourth section (7 items) comprised sociodemographic questions. The survey was anonymous (ie, no identifiable data were collected).

The survey was first reviewed by 2 physician researchers and 2 nurses who provided feedback for minor changes pertaining to the wording and order of the items. After improvements were made, the revised version of the questionnaire was pilot-tested with 5 patients with HF to ensure that the participants fully understood each question and were able to complete the survey within 15 to 20 minutes. No changes were made to the instrument after pilot-testing.

### Participant Recruitment and Data Collection

Eligible patients were approached during routine clinical appointments by research assistants who provided information about the study protocol and asked patients for informed consent to participate in the study. All interviews were conducted in private examination rooms at the outpatient HF clinic. Survey questions were read aloud by research assistants, and responses were captured and saved in a REDCap (Research Electronic Data Capture) database using study laptops. REDCap is a secure web-based application for building and managing questionnaires and databases for the collection and entry of research data [41].

### Statistical Analysis

Descriptive statistics (eg, frequency and percentage) were used to quantitatively analyze the adoption and use of mHealth technologies. Following the same approach as the Pew Internet Research study [42], we categorized the age groups as follows: <50 years, 50-64 years, and >65 years. Univariate analyses (chi-square tests) were used to examine associations between sociodemographic characteristics and mobile technology use across patients with HF, and the Cramér *V* (Φ_C_) was used to assess the strength of these associations. The Cramér *V* ranges from 0 to 1, where 0 to <0.10 is a negligible association, 0.10 to <0.20 is a weak association, 0.20 to <0.40 is a moderate association, 0.40 to <0.60 is a relatively strong association, 0.60 to <0.80 is a strong association, and 0.80 to 1.00 is a very strong association [43]. Content analysis was used to assess the attitudes toward and perceptions of the use of commercially available smartphones and wearable sensor devices for remote monitoring and delivery of HF self-care support via SMS text messages.

## Results

### Demographics

Over a 4-month period, 144 patients were approached and asked to participate in the survey. A total of 100 eligible patients (mean age 61.3, SD 12.3; range 29-93 years) agreed to participate, yielding a response rate of 69.4% (100/144). As shown in Table 1, most patients (63/100, 63%) were women, self-identified as Black or African American (61/100, 61%), were aged 50-64 years (49/100, 49%), had high school education or lower (65/100, 65%), and reported being retired or on disability (55/100, 55%). Of the 100 patients, only 49 (49%) reported their annual income. Of those who did report their annual household income, 53% (26/49) made <US $25,000 per year.

**Table 1:** Participant demographics (N=100).

Demographics		Values
**Gender, n (%)**		
	Male	37 (37)
	Female	63 (63)
**Race, n (%)**		
	White (non-Hispanic)	17 (17)
	Black or African American	61 (61)
	Hispanic or Latino	18 (18)
	Asian	1 (1)
	Other	3 (3)
Age (years), mean (SD)		61.32 (12.3)
**Age group (years), n (%)**		
	29-49	12 (12)
	50-64	49 (49)
	≥65	39 (39)
**Education, n (%)**		
	Lower than high school	18 (18)
	High school	47 (47)
	Undergraduate	26 (26)
	Graduate	9 (9)
**Employment status, n (%)**		
	Employed	28 (28)
	Unemployed	17 (17)
	Retired or on disability	55 (55)
**Income (US $), n (%)**		
	<25,000	26 (26)
	25,000-49,000	12 (12)
	50,000-74,999	6 (6)
	75,000-99,999	0 (0)
	>100,000	5 (5)
	Not sure or declined to respond	51 (51)

### Mobile Phone and Smartphone Ownership by Race and Age Group in Patients With HF

As shown in Table 2, most patients with HF owned a cell phone (93/100, 93%). The share of patients who owned a smartphone was 68% (68/100). Android smartphones (46/68, 67%) were owned at higher rates compared with iPhones (15/68, 22%) or other types of smartphones (7/68, 10%). Smartphone ownership was relatively higher in Black or African American participants (45/61, 74%) compared with White (9/17, 53%) and Latino or Hispanic participants (11/18, 61%). Adoption rates were twice as high in middle-aged adults (41/49, 84% ages 50-64 years) compared with people aged >65 years (16/39, 41%), illustrating a moderate association between age group and smartphone ownership (Φ_C_=0.351; *P*<.001). Employed participants were significantly more likely to own a smartphone (25/28, 89%) compared with those who were unemployed (12/17, 71%) or retired (31/55, 56%; Φ_C_=0.299; *P*=.001). Overall, the association between smartphone ownership and race (Φ_C_=0.273), education (Φ_C_=0.291), and income (Φ_C_=0.274) was moderate, whereas it was weak with gender (Φ_C_=0.122).

**Table 2:** Profile and smartphone ownership of participants across demographic groups (N=100)^a^.

Demographics			Participants with smartphone (n=68)^b^, n (%)		Participants with mobile phone but not smartphone (n=25)^c^, n (%)		Participants without mobile phone (n=7)^d^, n (%)		Φ_C_^e^			Chi-square (*df*)			*P* value	
**Gender**										0.122			1.5 (2)			.48
	Male	25 (68)		8 (22)		4 (11)										
	Female	43 (68)		17 (27)		3 (5)										
**Race**										0.273			14.9 (8)			.06
	White (non-Hispanic)	9 (53)		4 (24)		4 (24)										
	Black or African American	45 (74)		15 (25)		1 (2)										
	Hispanic or Latino	11 (61)		6 (33)		1 (6)										
	Asian	1 (100)		0 (0)		0 (0)										
	Other	2 (67)		1 (33)		0 (0)										
**Age (years)**										0.351			24.7 (4)			<.001
	29-49	11 (92)		1 (8)		0 (0)										
	50-64	41 (84)		8 (16)		0 (0)										
	≥65	16 (41)		16 (41)		7 (18)										
**Education**										0.291			16.9 (6)			.01
	Lower than high school	11 (61)		6 (33)		1 (6)										
	High school	29 (62)		15 (32)		3 (6)										
	Undergraduate	22 (85)		4 (15)		0 (0)										
	Graduate	6 (67)		0 (0)		3 (33)										
**Employment status**										0.299			17.9 (4)			.001
	Employed	25 (89)		1 (4)		2 (7)										
	Unemployed	12 (71)		2 (12)		3 (18)										
	Retired or on disability	31 (56)		22 (40)		2 (4)										
**Income (US $)**										0.277			7.5 (6)			.27
	<25,000	17 (65)		8 (31)		1 (4)										
	25,000-49,000	11 (92)		0 (0)		1 (8)										
	50,000-74,999	4 (67)		2 (33)		0 (0)										
	75,000-99,999	0 (0)		0 (0)		0 (0)										
	>100,000	5 (100)		0 (0)		0 (0)										
	Not sure	14 (54)		8 (31)		4 (15)										
	Declined to respond	17 (68)		7 (28)		1 (4)										

^a^Percentages add up to 100 horizontally.

^b^Mean age 57.49 (SD 11.05) years.

^c^Mean age 67.32 (SD 10.54) years.

^d^Mean age 77.14 (SD 7.65) years.

^e^Cramér *V*

Most patients who owned a smartphone (60/68, 88%) reported having a data plan that allowed them to access the internet on their phones. Most of these patients (44/60, 73%) had an unlimited data plan. There were no significant differences in data plan ownership between age groups (Φ_C_=0.143; *χ^2^*_4_=2.8; *P*=.59). However, people who reported being employed, retired, or on disability were significantly more likely to have an internet plan on their phone compared with those who reported being unemployed (Φ_C_=0.324; *χ^2^*_4_=14.3; *P*=.007).

### Text Messaging and HF Self-care

Of the 93 patients who owned a cell phone, 69 (74%) reported having an SMS text messaging plan. Most of them (61/69, 88%) had a plan that allowed them to send and receive an unlimited number of SMS text messages. Most patients with a cell phone (67/93, 72%) reported being somewhat comfortable or very comfortable with sending and receiving SMS text messages. Approximately half (43/93, 46%) said that they sent SMS text messages every day, whereas 30% (28/93) said they did not use the SMS text messaging feature. Frequency of and comfort with SMS text messaging use correlated with age. Patients aged ≥65 years reported sending SMS text messages less frequently (Φ_C_=0.327; *χ^2^*_6_=19.9; *P*=.003) and feeling less comfortable using the SMS text messaging feature than younger and middle-aged adults (Φ_C_=0.303; *χ^2^*_6_=17.1; *P*=.009). There was no significant relationship between SMS text messaging use and other demographic characteristics (eg, gender, race, education, or employment status). The patients were asked whether they would be interested in receiving weekly SMS text messages from the clinic to help them improve HF self-care and, if so, how many messages they would like to receive. A total of 68% (63/93) of patients said they would like to receive HF self-care messages. Most of them (34/63, 54%) said they would like to receive 1-2 messages per week. There were no significant differences in demographic characteristics (eg, age, gender, or race) between patients who expressed interest in receiving HF self-care messages on their cell phones and those who did not.

### Frequently Used Smartphone Features and Interest in Using mHealth Apps for HF Self-care

The patients were asked to indicate which smartphone features they used regularly in addition to calling. As shown in Table 3, SMS text messaging was the most frequently used smartphone feature, followed by internet browsing, mobile apps, social media, emails, and appointment scheduling. Age group was significantly associated with this type of use (Table 3). Older adults (aged ≥65 years) were less likely to use these features than younger and middle-aged adults.

**Table 3:** Frequently used smartphone features and popular heart failure (HF) parameters that patients would like to self-monitor using mobile apps and connected health devices, grouped by age (N=68).

Item		Overall sample, n (%)	29-49 years (n=11), n (%)	50-64 years (n=41), n (%)	≥65 years (n=16), n (%)	Φ_C_^a^	Chi-square (*df*)	*P* value
**Most frequently used smartphone features**								
	SMS text messaging	51 (75)	11 (100)	31 (76)	9 (56)	0.313	6.7 (2)	.04
	Internet browsing	46 (63)	11 (100)	25 (61)	7 (44)	0.366	9.1 (2)	.01
	Mobile apps	41 (60)	9 (82)	26 (63)	6 (38)	0.291	5.8 (2)	.06
	Social media	39 (57)	10 (91)	23 (56)	6 (38)	0.336	7.7 (2)	.02
	Email	33 (49)	10 (91)	17 (42)	6 (38)	0.374	9.5 (2)	.009
	Appointment scheduling	27 (40)	8 (73)	18 (44)	1 (6)	0.434	12.8 (2)	.002
**Most popular HF parameters that patients would like to self-monitor using mobile apps and connected devices**								
	Physical activity tracking	46 (68)	11 (100)	25 (61)	10 (63)	0.304	6.3 (2)	.04
	Blood pressure tracking	44 (65)	10 (91)	23 (56)	11 (69)	0.264	4.8 (2)	.09
	Medication tracking	40 (59)	10 (91)	23 (56)	7 (44)	0.304	6.3 (2)	.04
	Weight tracking	38 (56)	9 (82)	20 (49)	9 (56)	0.238	3.8 (2)	.15
	Diet tracking	36 (53)	8 (73)	19 (46)	9 (56)	0.192	2.5 (2)	.28
	Symptom tracking	35 (52)	8 (73)	20 (49)	7 (44)	0.191	2.5 (2)	.29
	Sleep tracking	31 (46)	7 (64)	17 (42)	7 (44)	0.160	1.8 (2)	.42
	Mood tracking	29 (43)	7 (64)	14 (34)	8 (50)	0.228	3.6 (2)	.17
	Blood sugar or diabetes	28 (41)	8 (73)	14 (34)	6 (38)	0.283	5.5 (2)	.07

^a^Cramér *V*.

Of the 68 patients who owned a smartphone, 15 (22%) reported using ≥1 mHealth app for self-monitoring of HF and other health-related data, 36 (53%) said that they did not use mHealth apps but would be interested in doing so in the near future, and 17 (25%) said that they were not interested at all in using mHealth apps. There was a significant relationship between education and current use of mHealth apps (Φ_C_=0.374; *χ^2^*_3_=9.5; *P*=.02).

Among those who reported using mHealth apps for HF self-care (15/68, 22%), frequently used apps included Fitbit, Apple Health, and Samsung Health for physical activity tracking (14/15, 93%); MyFitnessPal, Lose it!, and Eat24 for tracking of diet and low-sodium foods (11/15, 73%); and Apple Health or Samsung Health for monitoring of blood pressure (13/15, 87%) and glucose levels (6/15, 40%). Overall, as shown in Table 3, the most popular health parameters that patients with smartphones would like to self-monitor using mHealth apps were physical activity (46/68, 68%), blood pressure (44/68, 65%), medication intake (40/68, 59%), weight (38/68, 56%), and diet (36/68, 53%).

### Ownership of, Comfort With, and Interest in Other Mobile Devices and Technologies Among Patients With HF

Most participants reported having internet at home (73/100, 73%). Most (58/100, 58%) owned a PC or laptop, fewer participants owned a tablet (38/100, 38%), and even fewer owned a wearable activity tracking device (15/100, 15%). Of the participants who reported tablet ownership, Android or Apple tablets (26/38, 68%) were most frequently owned, whereas others reported Windows tablets (7/38, 18%), and some were unable to recall the tablet they owned (5/38, 13%). Most participants who owned tablets (n=38) reported being either comfortable or very comfortable (30/38, 79%) using a tablet. Of the 58 patients who owned a computer or laptop, 78% (45/58) reported being comfortable or very comfortable, and 22% (13/58) reported being somewhat or not comfortable using a computer. The interest in using wearables was high among patients who did not own an activity tracker (61/85, 72%). Also, the majority of study participants (76/100, 76%) reported willingness to transmit monitored data to their physician using mHealth devices.

## Discussion

### Principal Findings

In this study, we conducted a cross-sectional survey of 100 outpatients with HF (mean age 61.3, SD 12.25 years; 63/100, 63% women) at an urban academic hospital in the Midwest to investigate smartphone adoption and use of mHealth technologies for HF self-care. To our knowledge, this is the first study on mHealth adoption in a predominantly racial and ethnic minority HF population (61/100, 61% Black or African American and 18/100, 18% Hispanic or Latino). Our survey results showed that smartphone ownership was nearly ubiquitous (22/68, 85%) among younger and middle-aged patients with HF (aged 18-64 years) but significantly lower (16/39, 41%) among older adult patients (aged ≥65 years). The most common smartphone features used by the study participants were SMS text messaging (51/68, 75%), internet browsing (43/68, 63%), and mobile apps (41/68, 60%). The use of mHealth apps and wearable activity trackers (eg, Fitbits) for self-monitoring of HF-related parameters and management of symptoms was low (15/68, 22% and 15/100, 15%, respectively). However, 53% (36/68) expressed interest in using mHealth for HF self-care in the near future The most popular HF self-care measures the participants would like to monitor with the use of mHealth technologies were physical activity (46/68, 68%), blood pressure (44/68, 65%), and medication intake (40/68, 59%). Age, education, and employment status were significantly associated with smartphone ownership and mHealth uptake, whereas race, gender, and income were not.

### Comparison With Other Studies

This study complements and expands on the findings of previous reports on mHealth adoption among patients with HF [34-36]. In a 2018 survey of 50 patients (mean age 64.5, SD 8.3 years; 32% women) conducted at a US-based University Health System, Sohn et al [36] found that 90% of patients with HF owned a smartphone, 49% owned a tablet, and 29% had an activity tracker or smartwatch. However, the study population consisted of mainly White, male patients aged 50-80 years with significantly higher education levels compared with our study. Thus, the higher adoption rates of mHealth devices reported by Sohn et al [36] may not be generalizable to the overall HF population.

In another survey study that was published in 2016 and included 100 patients with HF (mean age 60, SD 15 years; 31% women, no race reported), Dorsch et al [34] found that most participants (79%) owned a computer, and 44% owned a “smart device.” Contrary to our study, Dorsch et al [34] did not provide a definition or classification of smart devices nor reported the proportion of participants who owned a smartphone. These differences prevent further comparisons between studies.

Cajita et al [35] conducted a cross-sectional survey study on 129 older adults with HF (mean age 71.3, SD 4.6 years; 26.4% women, 56.6% White) and found that 57.4% of the participants owned a smartphone. In our study, smartphone ownership among older adults with HF (aged ≥65 years) was 41% (16/39) and varied substantially by age: 58% (10/17) of 65- to 69-year-old patients owned smartphones, but that share dropped to 36% (4/11) among 70- to 74-year-old patients and 18% (2/11) among patients in their mid-70s and beyond. These findings are consistent with the Pew Research Center survey on technology use among seniors [44] and confirm that, similar to the general population, there exists a digital divide between younger and older adult patients with HF in terms of smartphone ownership. However, the share of older adult patients with HF who owned a smartphone seems to follow that of the general senior population, which has more than doubled since 2013 when smartphone adoption among them was just 18%, rose to 46% in 2018 [44], and now stands at 61% [45]. Thus, it is safe to assume that the digital divide between younger, middle-aged, and older adult patients with HF will further decrease in the near future, creating new opportunities for mHealth research and care delivery methods in older adult patients with HF.

In a nationally representative sample of 3248 adults with or at risk for cardiovascular disease from the 2018 Health Information National Trends Survey, Shan et al [46] reported a high prevalence of smartphone ownership (73%) but low uptake of mHealth apps (48%) and wearables (39%) to track progress toward a health goal. Younger age, higher education, and higher income were associated with greater odds of smartphone ownership and mHealth uptake. In our study, smartphone ownership was significantly correlated with educational attainment and employment but not with income. More specifically, similar to other surveys [42,44], we found that patients with HF who had a bachelor’s or advanced degree and those who worked were significantly more likely to own a smartphone than those who had not completed high school or were retired or on disability. However, contrary to other studies [42,44,46,47], we did not find a statistically significant correlation between income and smartphone ownership. This could be because half of our study participants declined to answer the question about income, resulting in participation bias and, as a result, our sample size was not large enough to reach statistical significance.

Racial and ethnic minorities that identified as Black or African American and Hispanic or Latino reported high smartphone ownership rates in our survey. This finding is consistent with a series of studies by the Pew Research Center among adults in the United States, which showed no statistically significant racial and ethnic differences in terms of smartphone ownership [42,45]. When accessing the internet, Black or African American and Latino or Hispanic adults may rely more on smartphones compared with White adults. Evidence illustrates that >25% of Hispanic and approximately 20% of Black or African American adults use smartphones as their only way to connect to the internet [48,49]. Black or African American and Hispanic or Latino patients with HF have the highest incidence and prevalence of HF, as well as worse clinical outcomes, compared with other racial and ethnic groups [1]. Thus, there is a great opportunity to capitalize on the high smartphone ownership and broadband access among racial and ethnic minority patients to enhance self-monitoring and management of HF through mHealth interventions that target key self-care behaviors for the most common reasons for HF readmissions (eg, medication and diet nonadherence) [50].

A key question when developing mHealth interventions to promote and facilitate self-management of chronic disease is which smartphone functionalities and features to use to deliver the intervention content to patients (eg, mobile apps, SMS text messages, and connected health devices). Our survey results indicate that the most frequently used feature among patients with HF was SMS text messaging. However, SMS text messaging is often overlooked by researchers compared with more sophisticated mHealth tools [51]. A recent systematic review and meta-analysis [27] found that, contrary to other conditions (eg, diabetes [29]), the efficacy of SMS text messaging for HF self-care remains largely underexplored. Virtually all trials of mHealth interventions to date have focused on testing technologically advanced interventions that involve remote patient monitoring with clinical feedback or interactive mHealth apps. However, it is important to consider that not all patients with HF are able to use such advanced technological solutions even if they have a smartphone and that this trend may further contribute to the digital divide and use gap that already exists between senior and younger patients. As *flip phones* and other nonsmart mobile phones are slowly phased out and replaced by smartphones, many senior patients find themselves with new and *shiny* technology that they do not know how to use. In fact, some older adult participants informed us during the survey that their children purchased the smartphones for them and that they did not know how to use apps or any other features besides calling and texting. This highlights 2 key aspects: (1) the importance of aligning mHealth interventions with the users’ preferences, accessibility, and technology skills and (2) the need to provide new users with adequate support and training toward the adoption of novel digital health technologies, especially when there is evidence supporting their efficacy.

Our survey results show that, although 60% (41/68) of patients with HF used mobile apps in their daily lives, the use of mHealth apps and wearable activity trackers for self-monitoring of HF-related parameters was significantly low (15/68, 22% and 15/100, 15%, respectively). Previous research [52] has shown that this may be because of lack of interest or awareness of the existence of HF apps [23,53], including concerns about mHealth apps collecting and sharing identifiable data publicly. Some patients in our study openly expressed concerns regarding the use of wearable activity trackers and apps, stating that mHealth data might be used in the future to influence their premiums or deny them insurance coverage and shared with third-party companies without the patients’ knowledge or previous consent. These concerns underpin the need to address privacy and security concerns with future HF interventions using mHealth technology. Notwithstanding these concerns, 53% (36/68) of study participants who owned a smartphone said that they would be interested in using mHealth apps for HF self-care if given the opportunity. This is consistent with the findings of Sohn et al [36] and highlights the prospect of more patients with HF experimenting with or using mHealth apps and wearables in the near future to self-monitor their condition.

Contrary to what one would expect, our data suggest that the most common HF-related measure patients were interested in monitoring was physical activity and not weight or HF symptom tracking, which are often monitored in mHealth or telehealth studies [27]. Previous research has shown that physical activity is the only nonpharmacological therapy that has proven to be effective in reducing mortality and hospitalizations in patients with HF [54,55]. Thus, physical activity is included as a recommendation in clinical guidelines and patient education materials [10,15,16]. Unlike other HF measures (eg, diet and symptoms), which require active monitoring, physical activity tracking is effortless with wearables as it can be monitored passively without requiring patients to perform any data entry [56]. This preference over mHealth technologies that perform passive monitoring is consistent with our observation in an ongoing clinical trial, in which we are seeing higher patient engagement with mHealth technologies that support passive monitoring and automated capturing of health data (eg, Fitbit wearable activity tracker) instead of active monitoring of symptoms, which requires manual data entry via an app [50]. Another reason that might explain this preference is that physicians and nurses often recommend the use of a pedometer or wearable activity tracker to patients to monitor and increase their steps. Wearable activity trackers and smartwatches have become popular over the years, and recent systematic reviews and meta-analyses have shown that they can help people increase their physical activity through self-monitoring and goal setting [57-59]. Other connected health devices such as smart weight scales and blood pressure monitors are less popular.

### Limitations

There are several limitations that need to be considered in the interpretation of the results. First, the study was conducted at a single site in a large urban academic health system. Therefore, the results may not be generalizable to patients with HF who live in rural areas. Second, our sample comprised more women than men (63/100, 63% vs 37/100, 37%), although our HF clinic sees approximately 53% male patients with HF per year, which is similar to most data aggregates regarding the prevalence of HF [1]. It is likely that female patients were more receptive to participating in the survey, thus leading to a slight skew in our study cohort. However, we believe that this imbalance did not affect our study results, as previous studies have shown that there are no significant differences in smartphone and mHealth adoption between men and women [34,46].

Third, unlike other studies [36,60], we did not collect patients’ HF clinical characteristics (eg, New York Heart Association Class or left ventricular ejection fraction) to explore potential relationships between HF severity and smartphone adoption or use. A similar study found that these characteristics were not associated with the adoption or perceptions of mHealth [36]. Therefore, we do not believe that the absence of these data had a major impact on the interpretation of our results. Fourth, our participant population did not include people with major cognitive impairments and comprised only English-speaking individuals because of limited resources. Finally, we did not assess digital literacy among smartphone owners. Despite relatively high ownership, it is likely that some participants were limited in their ability to use features such as mHealth apps, email, or social media apps.

### Implications for Practice and Research

This study contributes to the mHealth literature by demonstrating smartphone ownership and mobile app use among a predominantly racial and ethnic minority population in an urban clinical setting in the Midwest. Smartphone ownership and use appear to cut across gender and race. We identified that participants used SMS text messaging and internet browsing at much higher rates than other more sophisticated features. Many mHealth interventions are built around complex remote monitoring apps; however, SMS text messaging interventions in HF remain underexplored. Health disparities exist among racial and ethnic minority populations, but we demonstrated a potentially innovative way to promote patient self-management because of high ownership and use of SMS text messaging. These factors may help address the disparate outcomes between racial groups with tailored SMS text messaging interventions. Furthermore, hospitals would be an important setting for promoting smartphone technology use to their patients. For example, patients may be trained to self-monitor symptoms, weight, or steps before discharge to prevent rehospitalizations. SMS text messages and mobile apps connect patients and providers to answer questions, address concerns, and provide guidance or education. We know that self-care is the key to improving outcomes in HF by leveraging existing technology. The results of this survey can guide future research to meet patients at their comfort level with smartphone technology rather than where we hope they would be.

Leveraging a scalable solution using smartphone technology in racial and ethnic minority populations would promote engagement and allow researchers to design future mHealth interventions. These important findings can support the creation of future interventions that rely on mHealth to monitor physical activity, blood pressure, weight, and medication adherence.

## Appendix

Multimedia Appendix 1 Survey of smartphones and wearable tracking devices in patients with heart failure.

## Abbreviations

HF: heart failure
mHealth: mobile health
REDCap: Research Electronic Data Capture
