# Supplementary material for: Smartphone Ownership and Interest in Mobile Health Technologies for Self-care Among Patients With Chronic Heart Failure: Cross-sectional Survey Study
Source: JMIR Cardio. 2022 Jan 14;6(1):e31982. doi: 10.2196/31982 (PMC8800088; doi:10.2196/31982)
Supplement: Multimedia Appendix 1 [file cardio_v6i1e31982_app1.docx]

**Appendix A: Survey of Smartphone and wearable tracking devices in Heart Failure patients**

1. **Mobile phone ownership and use**
2. Do you own a cell phone?

- Yes
- No → Please proceed to Question 19

1. How often do you use your cell phone?

- Frequently (a few times a day)
- Sometimes (a few times a week)
- Rarely (a few times a month)
- Never

1. Do you have a text-messaging plan with your phone company that allows you to send and receive text-messages?

- Yes
- No
- I don’t know

1. Select the option that best describes your text messaging service plan:

- Unlimited text-messaging
- Limited number of free text-messages per month (specify: ______)
- Pay-as-you-go
- I don’t know

1. How comfortable are you sending and receiving text-messages?

1 - Very comfortable

2 - Somewhat comfortable

3 – A little comfortable

4 – Not comfortable at all

1. How often do you send text-messages?
   - Everyday
   - Just a few times each week
   - Just a few times each month
   - I don’t text
2. Would you be interested in receiving text-messages on your cell phone from your doctor (e.g. educational or motivational messages and medication reminders) to help you improve self-management of your condition?
   - Yes
   - No → Please proceed to Question 9
3. How many text-messages would you like to receive on average per week from your care provider to help you improve self-management of your condition?
   - 1-2 messages per week
   - 3-4 messages per week
   - 5-7 messages per week
   - 8-10 messages per week
   - Other (Please specify:________)
4. **Ownership and use of a smartphone**
5. Some cell phones are called “smartphones” because of certain features they have. Is your cell phone a smartphone?

- Yes, it is a smartphone
- No, it is not a smartphone → Please proceed to Question 19
- Not sure / Don’t know → Please proceed to Question 19

1. What type of smartphone do you have?

- iPhone
- Samsung
- HTC
- Blackberry
- LG
- Motorola
- Other (please specify): ___________

1. Do you have a data plan that allows you to browse the Internet and send/receive emails using your cell phone?

- Yes
- No → Please proceed to Question 13
- I don’t know → Please proceed to Question 13

1. Select the option that best describes your data service plan:

- Unlimited data
- Limited number of data per month. Amount: _____
- Other: _______

1. Which of the following features do you use on your smartphone? Select all that apply.
   - Text-messaging
   - Information seeking on the Internet
   - Email
   - Mobile apps (applications)
   - Social media (e.g. Facebook, Twitter)
   - Scheduling (e.g. appointments, to-do lists)
2. How comfortable are you using a Smartphone?

- Very comfortable
- Comfortable
- Somewhat comfortable
- Not comfortable

1. Are you using any mobile applications that help you self-monitor and manage your health?

- Yes → Please proceed to Question 17
  - No

1. Would you like to be able to use one or more applications on your phone to help you self-manage your condition?
   - Yes → Please proceed to Question 18

- No → Please proceed to Question 19

1. Which mobile apps are you currently using to self-manage your condition?

_____________________________________________________________

1. Which of the following parameters are you monitoring/would you like to monitor using your smartphone?
   - Medication management
   - Blood pressure
   - Symptoms
   - Weight
   - Diet, food, calorie counter
   - Physical activity (e.g. steps) and/or exercise
   - Blood sugar or diabetes
   - Sleep
   - Mood
   - Other - Specify:_________________
2. **Ownership and use of other mobile/computing devices**
3. Do you own a tablet (e.g. iPad)?

- Yes
- No → Please proceed to Question 22

1. What type of tablet do you have?

- Android
- Apple (iOS)
- Windows
- Other (Please specify):
- Don’t know

1. How comfortable are you using a Tablet?

- Very comfortable
- Comfortable
- Somewhat comfortable
- Not comfortable

1. Do you own a computer (PC or laptop) at home?

- Yes
- No

1. How comfortable are you using a computer?

- Very comfortable
- Comfortable
- Somewhat comfortable
- Not comfortable

1. Do you have Internet at home?

- Yes
- No

1. Have you ever used or are you currently using any wearable tracking devices such as Fitbit to self-monitor your physical activity or sleep?
   - Yes
   - No
2. Would you be interested in using a wearable device such as Fitbit to self-monitor your physical activity, heart rate, sedentary time, and sleep quality?
   - Yes
   - No
3. Would you be willing to transmit your health monitored data to your doctor remotely using a smartphone, tablet, or computer?
   - Yes
   - No
   - Other (specify):
4. **Demographic information**
5. What is your gender?

- Male
- Female
- Other

1. How old are you? ___________
2. Which one or more of the following would you say is your race?

- White (non-Hispanic)
- Black or African American
- Hispanic or Latino
- Asian
- Native Hawaiian or Other Pacific Islander
- Native American or American Indian or Alaska Native
- Other

1. What is your highest level of education completed?

- No schooling completed
- Elementary school
- Some high school, no diploma
- High school
- College Degree (e.g. Associate or Bachelor’s degree)
- Master’s Degree
- Doctorate degree
- Other

1. What is your current work situation?
   - Employed full-time
   - Employed part-time
   - Unemployed
   - Retired
   - Disability
2. Which of the following categories best describes your total combined family income for your household for the past 12 months? This should include income (before taxes) from all sources (e.g. wages, social security, disability and/or veteran’s benefits, unemployment benefits, and so on).

- Less than $25,000
- Between $25,000 and $49,999
- Between $50,000 and $74,999
- Between $75,000 and $99,999
- Between $100,000 and $149,999
- More than $150,000
- Don’t Know/Not sure
- Decline to respond

1. Would you be interested in being contacted by phone or email regarding a future study that we will conduct at UIC to evaluate the effectiveness of using smartphone-based telemonitoring on self-management of heart failure?
   - Yes
   - No → Exit the survey
2. Please enter the following information

First Name:

Last Name:

Phone number:

Email:
